# The dynamics of deltamethrin resistance evolution in *Aedes albopictus* has an impact on fitness and dengue virus type-2 vectorial capacity

**DOI:** 10.1186/s12915-023-01693-0

**Published:** 2023-09-13

**Authors:** Yijia Guo, Ke Hu, Jingni Zhou, Zhensheng Xie, Yijie Zhao, Siyu Zhao, Jinbao Gu, Xiaohong Zhou, Guiyun Yan, Anthony A. James, Xiao-Guang Chen

**Affiliations:** 1 Department of Pathogen Biology, Institute of Tropical Medicine, School of Public Health, Southern Medical University, Guangzhou, China; 2 Program in Public Health, University of California, Irvine, Irvine, CA USA; 3 Department of Microbiology & Molecular Genetics, University of California, Irvine, CA 92697-4025 USA; 4 Department of Molecular Biology & Biochemistry, University of California, Irvine, CA 92697-3900 USA; 5 Irvine, USA

**Keywords:** Target-site resistance, Metabolic resistance, Selection, Pyrethroid, Vector competence

## Abstract

**Background:**

Worldwide invasion and expansion of *Aedes albopictus*, an important vector of dengue, chikungunya, and Zika viruses, has become a serious concern in global public health. Chemical insecticides are the primary means currently available to control the mosquito populations. However, long-term and large-scale use of insecticides has selected for resistance in the mosquito that is accompanied by a genetic load that impacts fitness.

**Results:**

A number of laboratory strains representing different resistance mechanisms were isolated and identified from laboratory-derived, deltamethrin-resistant *Ae. albopictus* recovered in previous work. Resistance levels and fitness costs of the strains were evaluated and compared to characterize the evolution of the resistance genotypes and phenotypes. The heterozygous F1534S mutation (1534^F/S^) in the *voltage gated sodium channel* (*vgsc*) gene product (VGSC), first detected in early stages of resistance evolution, not only confers high-level resistance, but also produces no significant fitness costs, leading to the rapid spread of resistance in the population. This is followed by the increase in frequency of homozygous F1534S (1534^S/S^) mosquitoes that have significant fitness disadvantages, prompting the emergence of an unlinked I1532T mutation with fewer side effects and a mating advantage better adapted to the selection and reproductive pressures imposed in the experiments. Metabolic resistance with no significant fitness cost and mediating a high-tolerance resistance phenotype may play a dominant role in the subsequent evolution of resistance. The different resistant strains had similar vector competence for dengue virus type-2 (DENV-2). Furthermore, a comparative analysis of vectorial capacity revealed that increased survival due to deltamethrin resistance balanced the negative fitness cost effects and contributed to the risk of dengue virus (DENV) transmission by resistant populations. The progressive evolution of resistance results in mosquitoes with both target-site insensitivity and metabolic resistance with lower fitness costs, which further leads to resistant populations with both high resistance levels and vectorial capacity.

**Conclusions:**

This study reveals a possible mechanism for the evolution of deltamethrin resistance in *Aedes albopictus*. These findings will help guide practical strategies for insecticide use, resistance management and the prevention and control of mosquito-borne disease.

**Supplementary Information:**

The online version contains supplementary material available at 10.1186/s12915-023-01693-0.

## Background

*Aedes albopictus* (Skuse) is one of the most invasive species in the world [1] as well as an important vector for mosquito-borne diseases such as dengue fever, chikungunya fever and zika [2–4]. Dengue fever is the most prevalent arboviral disease globally, with 3.9 billion people in 128 countries or regions at risk [5]. Currently, due to the lack of reliable drugs or vaccines, the most effective way to prevent dengue virus (DENV) transmission is to use chemical insecticides to control mosquito populations. The decades-long intensive use of pyrethroid insecticides, the most commonly used class for adult mosquito control [6], has resulted in the evolution of resistance in mosquitoes across the globe [7–9].

Recognized mechanisms of pyrethroid resistance include target-site insensitivity that affects the binding affinity between the chemicals and their targets, and includes knockdown resistance (*kdr*), caused by point mutations in the *voltage-gated sodium channel* (*vgsc*) gene, and metabolic detoxification of insecticides prior to them reaching their targets (metabolic resistance) [10–13]. However, the role of the two mechanisms in resistance evolution is poorly understood due to the complex history of insecticide use in the field and the different resistance phenotypes and genetic backgrounds of mosquito populations. In previous work [14], a laboratory-derived resistance strain (Lab-R) was established by selecting with deltamethrin, the most commonly-deployed pyrethroid [15], for 30 generations (Lab-R30) from the laboratory susceptible strain (Lab-S). An F1534S substitution in the VGSC was detected first after six generations of selection and was sufficient for the deltamethrin resistance phenotype. Subsequently, an I1532T substitution in VGSC and metabolic resistance mediated by cytochrome P450 monooxygenase (P450s), choline/carboxylesterases (CCEs) and glutathione-S-transferases (GSTs) were detected at the 24^th^ generation.

Mutations that confer insecticide resistance also impose a genetic load that results in a fitness cost in the mosquitoes [16]. Fitness costs can manifest in numerous ways, including disadvantages in development, reproduction, survival and behavior, which reduce the vitality of resistant mosquitoes. The two main resistance mechanisms, target-site insensitivity and metabolic, are accompanied by reductions in fitness [16], but costs associated with a specific resistance mechanism are largely unknown and their impact on the evolution of resistance is even more unclear. It is worth noting that *kdr* mutations may influence the transmission of odorant signals towards higher brain regions by prolonging the inactivation of VGSC [17], and have been reported to change mosquito behavior in a manner that impacts male mating competitiveness and female host-seeking ability [18–20]. These behavioral costs appear to directly affect the competitive advantage of the resistant mosquitoes but more work needs to be done to understand this.

Insecticide resistance is responsible in part for the ineffective control of vector-borne diseases because it hinders the decline of vector density. However, fitness disadvantage associated with insecticide resistance, including shortened life span [21], reduced competence [21, 22] or behavioral changes [18], can result in reduced vector densities and potentially lower the risk of transmission [23]. Hence, the dual impact of the loss of insecticide effectiveness and fitness costs fosters efforts to identify and evaluate factors to determine the combined impact on the evolution of resistance and the subsequent transmission of vector-borne disease pathogens. In particular, the effect of the development of resistance on the vectoring abilities of the mosquito is important [24].

Vectorial capacity (VC), which describes the capacity of a vector population to transmit a pathogen, takes into account tripartite interactions among the host, pathogen, and vector, and was refined by Garret-Jones [25] using the classical Equation (1) that was improved subsequently to Equation (2) [26]. Equation (2) includes the following parameters: the ratio of mosquitoes to humans (*m*, density); human biting rates (*a*); vector competence (*b*); the average lifespan of a mosquito (1/*g*); and the extrinsic incubation period (EIP, *N* days). The equation has been applied in field studies in the exploration of epidemic causes and trends of vector-borne diseases [23, 27, 28]. The equation also has been used in laboratory studies to assess the effects of factors on the transmission of pathogens, including arboviruses [29–31]. However, the application of VC has had historical problems related to lack of representative samples and the application of comparable experimental data [25]. It is necessary to further refine the definition of parameters to promote the comparability of research factors.

1 $${VC=\frac{m{a}^{2}{p}^{N}}{-\mathrm{ln}(p)}}$$

2 $${VC=\frac{m{a}^{2}{b{e}^{-gN}}}{g}}$$

A metanalysis of previous work examining the link between the development of resistance and vectorial capacity found that there were examples of enhancement, impairment or no effect [24]. In this study, we used several laboratory-derived resistant *Ae. albopictus* strains to further explore the role of target-site insensitivity (F1534S/I1532T) and metabolic resistance in the evolution of deltamethrin resistance. We determined the resistance levels and fitness costs contributed by the two resistance mechanisms and also compared the behavioral costs and vector competence to DENV-2. Furthermore, the vectorial capacity equation was used to evaluate the comprehensive impact of the evolution of deltamethrin resistance on the potential transmission of dengue viruses.

## Results

### Establishment of laboratory strains representing different resistance mechanisms

**Fig. 1:**
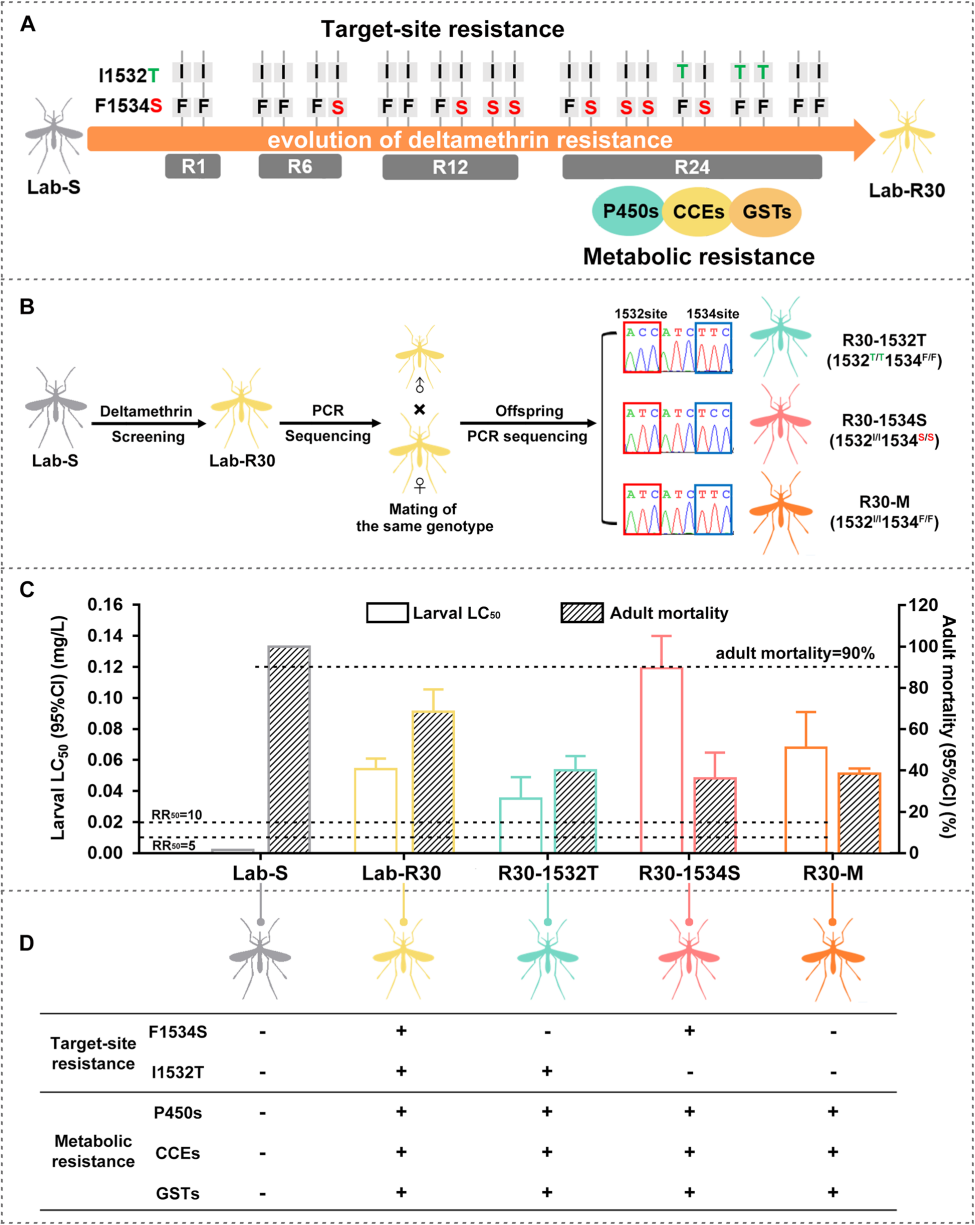
Evolutionary mechanism of deltamethrin resistance in *Aedes albopictus*. **A** An *Ae. albopictus* laboratory deltamethrin-resistant strain (Lab-R) was derived successfully from the laboratory susceptible strain (Lab-S) in previous work [14]. An F1534S substitution (target-site resistance, F/S) in the *voltage-gated sodium channel* (*vgsc*) gene was the first event detected during the emergence of resistance. A second mutation, I1532T (I/T), was subsequently detected accompanied by the emergence of increased metabolic enzyme activity, including cytochrome P450 monooxygenases (P450s), choline/carboxylesterases (CCEs) and the glutathione-S-transferase (GSTs), also likely involved in resistance. The different selected generations are the 6^th^ generation (R6), the 12^th^ generation (R12), the 24^th^ generation (R24) and the 30^th^ generation (Lab-R30) [14]. **B** Isolation of sub-strains from the Lab-R30 strain. The genotypes of unmated individuals in Lab-R30 were identified by sequencing PCR products. The unmated individuals with the same genotypes were crossed to obtain R30-1532T (1532^T/T^1534^F/F^), R30-1534S (1532^I/I^1534^S/S^) and R30-M (1532^I/I^1534^F/F^) strains. **C** Larval and adult bioassays of deltamethrin resistance in different strains (*n* = 10, 20–30 adults per strain in each experiment). Error bars represent 95% CIs. **D** Target-site resistance (I1532T/F1534S mutations) and metabolic resistance involved in the evolution of resistance to deltamethrin

During the deltamethrin screening over 30 generations in previous work [14], an F1534S substitution was first observed at the early stages of resistance evolution, followed by an I1532T substitution and metabolic resistance (P450s, CCEs and GSTs) detected at later stages (Fig. 1A). The F1534S mutant heterozygotes (F1534S’) and homozygotes (F1534S) obtained in the previous study [14] with no detectable metabolic resistance (Table 1, Additional File 1: Fig. S1) were used to evaluate the early contribution of the F1534S mutation to resistance evolution. In order to evaluate the contribution of target-site insensitivity (I1532T/F1534S) and metabolic changes to the later stage of resistance evolution, we successfully isolated three sub-strains, R30-1532T, R30-1534S and R30-M, from Lab-R30 (Fig. 1B), which represent different resistance mechanisms (Table 1, Fig. 1D). The WHO classification criteria [32] defines high level resistance status as a larval resistance ratio (RR_50_), RR_50_ > 10, and adult mortality < 90%. Larval and adult bioassay results revealed that Lab-R30 and the three sub-strains had a high level of deltamethrin resistance, with R30-1534S the highest (RR_50_ = 59.5, adult mortality = 36.2%), followed by R30-M (RR_50_ = 34.0, adult mortality = 38.6%) and Lab-R30 (RR_50_ = 27.0, adult mortality = 68.6%), and that of R30-1532T was the lowest (RR_50_ = 17.5, adult mortality = 40.0%) (Fig. 1C). R30-1532T with metabolic resistance is homozygous for I1532T, R30-1534S with metabolic resistance is homozygous for F1534S, and R30-M is mediated by metabolic resistance with no mutations detected at the 1532/1534 loci (Fig. 1D, Additional File 1: Fig. S1).

**Table 1:**
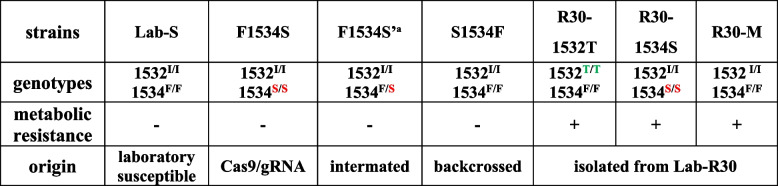
Control and deltamethrin-resistant *Ae. albopictus* strains used and selected

^a^Heterozygous line

*Abbreviations*: *F* phenylalanine, *I* Isoleucine, *S* Serine, *T* Threonine (black letters are the wild-type genotype and red- and green- colored are the mutations)

### Fitness costs generated in the evolution of deltamethrin resistance in *Aedes albopictus*

**Fig. 2:**
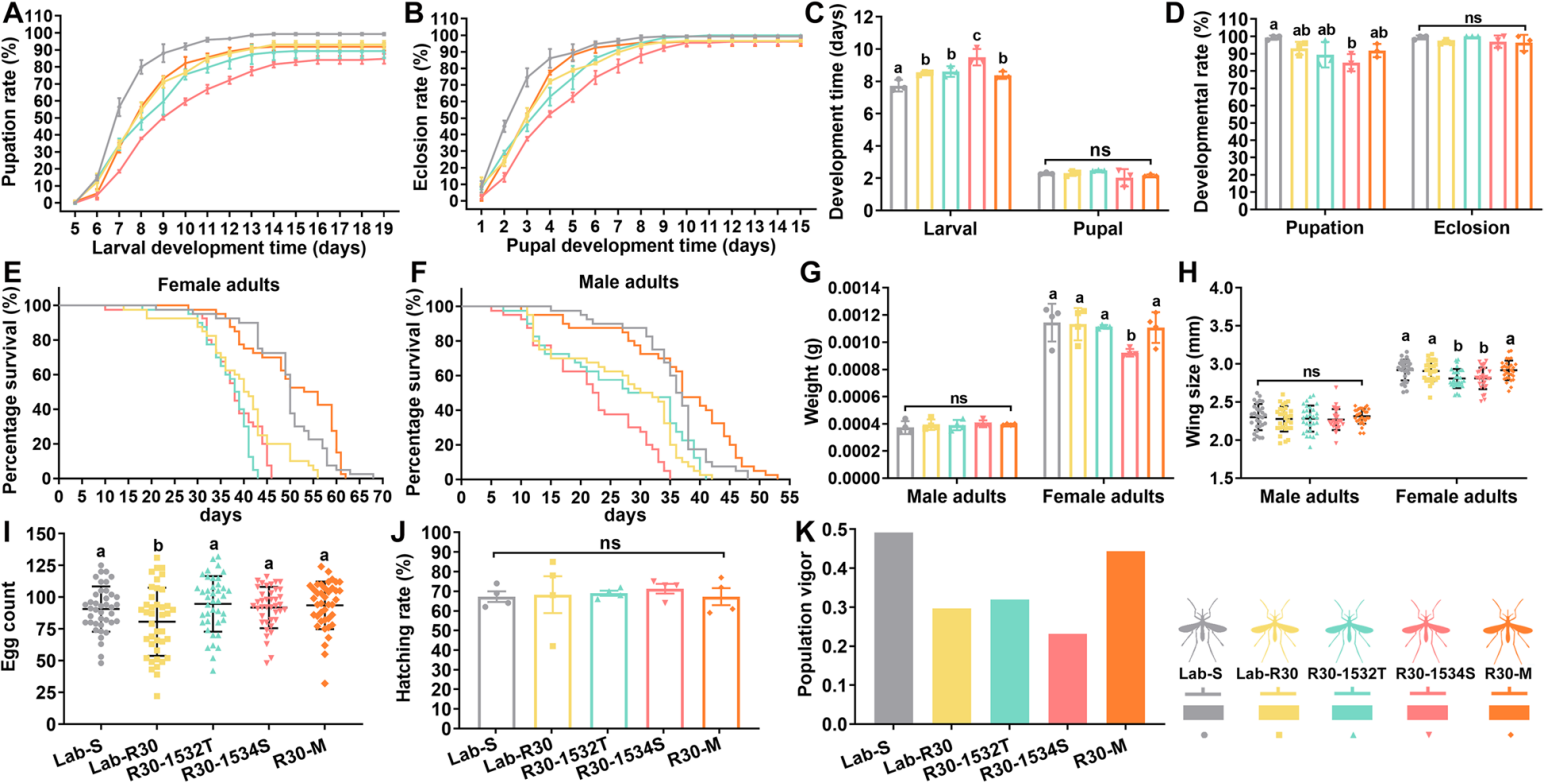
Life history analyses of different strains of *Aedes albopictus*.** A** Temporal trend of pupation rate. **B** Temporal trend of eclosion rate. **C** Larval and pupal developmental time. **D** Pupation rate of larvae and eclosion rate of pupae. **A-D** *n* = 3, 50 newly hatched larvae per strain in each experiment. **E** Survival curves of female adults (*n* = 40). Comparison of survival curves among different strains and Lab-S based on the Log-rank test: Lab-R30 vs Lab-S (*χ*^2^ = 21.42, *df* = 1, *P* < 0.0001), R30-1532 T vs Lab-S (*χ*^2^ = 63.18, *df* = 1, *P* < 0.0001), R30-1534S vs Lab-S (*χ*^2^ = 48.55, *df* = 1, *P* < 0.0001), R30-M vs Lab-S (*χ*^2^ = 2.02, *df* = 1, *P* = 0.1553). **F** Survival curves of male adults (*n* = 40). Comparison of survival curves among different strains and Lab-S based on the Log-rank test: Lab-R30 vs Lab-S (*χ*^2^ = 15.76, *df* = 1, *P* < 0.0001), R30-1532 T vs Lab-S (*χ*^2^ = 6.96, *df* = 1, *P* = 0.0083), R30-1534S vs Lab-S (*χ*^2^ = 50.89, *df* = 1, *P* < 0.0001), R30-M vs Lab-S (*χ*.^2^ = 3.533, *df* = 1, *P* = 0.0602). **G** Weight of adults (*n* = 4, 8 pooled adults each experiment). **H** Wing size of adults (*n* = 30). **I** Fecundity of female adults (*n* = 40). **J** Hatching rate per 100 eggs (*n* = 4, 100 eggs per strain). **K** Comprehensive quantification of fitness cost. Population vigor = (number of eggs*hatching rate*pupation rate*eclosion rate*average life span of adults*average weight of adults*average wing length of adults) / (larval development time + pupal development time). Ranking of fitness cost of strains: R30-1534S > Lab-R30 > R30-1532 T > R30-M > Lab-S. The results are represented as the mean ± SD. ns: not significant. Different lowercase letters represent significant differences among sample groups a, b, and c (*P* < 0.05)

The fitness costs of resistant strains were evaluated (Fig. 2, Additional File 1: Tables S1 and S2). R30-1534S with the highest resistance level showed significant fitness costs, with the longest growth period (Fig. 2A-C) and the lowest pupation rate (Fig. 2D), but the pupal development time (Fig. 2C) and eclosion rate (Fig. 2D) were not significantly different from the other strains. The larval developmental time of Lab-R30, R30-1532 T and R30-M also was significantly higher than that of Lab-S (Fig. 2C). The life spans (longevity) of Lab-R30, R30-1534S and R30-1532 T female and male adults were significantly shorter than that of Lab-S (Fig. 2E, F). No significant differences were observed in body weight and wing-lengths of male adults among the five strains (Fig. 2G, H), but the female wing-lengths of R30-1534S and R30-1532 T, as well as the female body weight of R30-1534S, were significantly smaller (Fig. 2G, H). Lower fecundity compared to controls of Lab-R30 was observed (Fig. 2I), and there was no difference in egg hatchability among the strains (Fig. 2J). According to the quantitative analysis of fitness cost, the fitness cost of R30-1534S is the highest, followed in order by Lab-R30, R30-1532 T, R30-M (Fig. 2K). These results confirm that resistant mosquitoes have significant loads that results in lower fitness when compared to wild-type mosquitoes.

### Homozygous mutant F1534S mosquitoes have high level deltamethrin resistance and significant fitness costs

Based on the results of significant fitness cost of R30-1534S, we investigated the role of F1534S in the early process of resistance evolution. The susceptible reference strain Lab-S (1534^F/F^), gene-edited strain F1534S (1534^S/S^), mutant heterozygote F1534S’ (1534^F/S^) and mutant restored strain S1534F (1534^F/F^) constructed previously[14], were used to assess fitness impacts (Additional File 1: Figs. S2, Tables S1 and S2). No significant differences were observed in the pupal development time (Additional File 1: Fig. S2D), developmental rate (Additional File 1: Fig. S2E), weight of male adults (Additional File 1: Fig. S2H), wing length (Additional File 1: Fig. S2I) and egg hatchability (Additional File 1: Fig. S2K) among the four strains. The life history traits of S1534F with restored susceptibility to deltamethrin were consistent with those of the Lab-S control (Additional File 1: Fig. S2L). Similar to R30-1534S, homozygous F1534S mosquitoes showed the most significant fitness cost (Additional File 1: Fig. S2L) with the longest growth period, development (Additional File 1: Fig. S2B, C), longest larval development time (Additional File 1: Fig. S2D), shortened life span of adults (Additional File 1: Fig. S2F, G), lighter body weight of female adults (Additional File 1: Fig. S2H) and decreased fecundity (Additional File 1: Fig. S2J). Heterozygous F1534S’ mosquitoes with high level resistance were not disadvantaged significantly (Additional File 1: Fig. S2L), likely contributing to its rapid spread in the population at the early stage of resistance evolution.

### I1532T mutations with lower fitness costs have an advantage in the evolution of resistance in *Aedes albopictus*

#### The binding affinity of deltamethrin to mutant F1534S VGSC protein is lower than that of the I1532T mutation

**Fig. 3:**
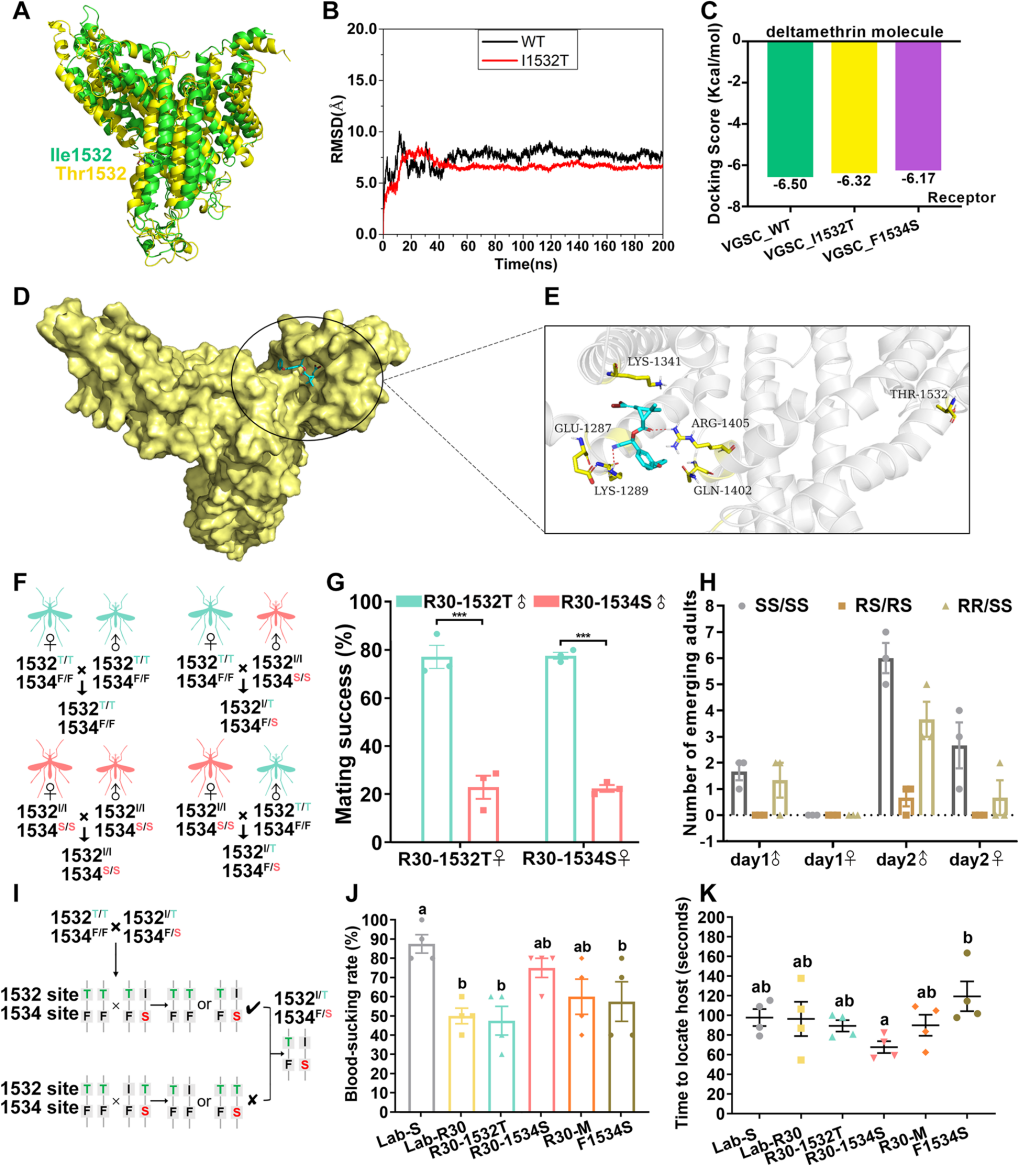
The late-occurring I1532T mutation has a stronger selective advantage than the F1534S mutation. **A** Structural and functional changes in VGSC caused by I1532T. Final stable structure at 200 ns simulation time of VGSC_WT superposed with VGSC_I1532T. VGSC_WT is colored in green and VGSC_I1532T in yellow. The residue Ile1532 and mutated Thr1532 are shown in stick view with corresponding color. **B** System flexibility analysis of VGSC_WT and VGSC_I1532T. The root-means-square deviation (RMSD) of the backbone of VGSC_WT is less than 8.0 angstrom and that of VGSC_I1532T is less than 7.0 angstrom, and the system achieves equilibrium within the simulation time. **C** The docking scores of deltamethrin molecules with VGSC_WT [14], VGSC_F1534S [14] and VGSC_ I1532T proteins. **D** The binding model of deltamethrin on the molecular surface of VGSC_I1532T. Deltamethrin is colored in cyan, and the molecular surface of protein is colored in pale yellow. **E** The 3D binding mode of deltamethrin interaction with VGSC_I1532T. Deltamethrin is colored in cyan, the surrounding residues in the binding pockets are colored in yellow, and the backbone of the receptor is depicted as a white cartoon with transparency. **F** Male mating competitiveness in different strains. Genotypes of offspring from female adults of different strains after successful mating with male adults from different strains. **G** Mating success rate between R30-1532 T and R30-1534S (*n* = 3). ***P < 0.001. **H** Genotypes of adults on the first and second day of emergence in Lab-R30 (*n* = 3). Homozygous wild-type is represented as SS, heterozygous as RS and homozygous mutant as RR. SS/SS: 1532^I/I^1534^F/F^, RS/RS: 1532^I/T^1534^F/S^, RR/SS: 1532^ T/T^1534^F/F^. **I** I1532T and F1534S mutations in *vgsc* gene in Lab-R strain were located on different chromosomes of a homologous pair. **J** The percentage of starved females from each strain successfully taking a human blood meal (*n* = 4). **K** Mean time for starved females from each strain to locate a host (*n* = 4). Different lowercase letters, a and b, represent significant differences (*P* < 0.05). The results in **G**, **H**, **J** and **K** are represented as the mean ± SEM

The structure of VGSC_I1532T was simulated on the basis of VGSC_WT constructed in a previous study [14] in order to explore the difference of the effects of the two mutations on deltamethrin resistance. The final stable structure and root-means-square deviation (RMSD) of the VGSC_I1532T backbone (Fig. 3A, B) showed that the overall conformations of VGSC_ I1532T (RMSD = 4.143 Å) was more similar to VGSC_WT than VGSC_F1534S (RMSD = 5.383 Å) [14]. The docking scores showed that the combination of deltamethrin with VGSC_WT was the best, followed by the combination with VGSC_I1532T, with the weakest being VGSC_F1534S (Fig. 3C). The nitrogen (cyan group) and oxygen atoms in deltamethrin, regarded as hydrogen acceptors, form hydrogen bonds with the side-chain nitrogen atom of Lys1289 and Arg1405 in VGSC_I1532T (Fig. 3D, E). Deltamethrin binds to the VGSC_WT by interacting with Arg1405 and Lys1341 via hydrogen bonding and forming *H-π* stacking with the sidechain of Gln1402 [14]. Only one hydrogen bond was formed between deltamethrin and Arg1405 when binding to the VGSC_F1534S [14]. These interactions of deltamethrin with VGSC proteins are consistent with the docking scores and resistance bioassays described above, supporting the conclusion that the lower resistance level induced by the I1532T mutation may be due to the different effects of the two mutations on the affinity of VGSC and deltamethrin.

#### Male adults with I1532T have a higher mating advantage than that of F1534S males

In the evolution of deltamethrin resistance in Lab-R, the frequency of I1532T increased after first detection while that of F1534S began to decrease [14]. The effects of the two mutations on the mating ability of males were evaluated as a possible evolutionary mechanism of the frequency changes in the resistant population. When adult male R30-1534S and R30-1532T strains were evaluated in competition (Fig. 3F), the latter had a significant mating advantage (Fig. 3G). In addition, the genotypes of Lab-R30 male adults that emerged on the first and second days were 1532^I/I^1534^F/F^ and 1532^T/T^1534^F/F^, and the genotypes of female adults emerging on the second day also were dominated by 1532^I/I^1534^F/F^ and 1532^T/T^1534^F/F^ (Fig. 3H). This temporal shift in eclosion is consistent with the development time in fitness cost (Fig. 2), supporting the conclusion that R30-1532T male adults had the mating advantage in the resistant population not only because of faster emergence, but also because of their significant mating competitiveness.

#### Resistance has an adverse effect on host seeking behavior

All starved females that located the host successfully took a blood meal. The feeding rate within five minutes of Lab-S was the highest (Fig. 3J, *χ*^2^ = 20.907, *df* = 5,* P* = 0.001), and the time for locating the host was not significantly different from that of other strains (Fig. 3K, F= 2.113, *df* = 5, *P* = 0.111). Among the resistant strains, R30-1534S had the highest feeding rate and the least elapsed time, while Lab-R30 and R30-1532T had lower feeding rates (Fig. 3J). The host-seeking behavior of F1534S was significantly prolonged with the lower feeding rate and the longest elapsed time (Fig. 3J, K; Additional File 1: Table S3).

#### The I1532T mutation emerged independently from F1534S during the evolution of deltamethrin resistance

Adult mosquitoes with 1532^T/T^1534^F/F^ and 1532^I/T^1534^F/S^ genotypes were mated and the genotypes of resulting progeny (F1) screened (Fig. 3I). If the two mutants were on different chromosomes, the progeny genotypes would be 1532^ T/T^1534^F/F^ and 1532^I/T^1534^F/S^. If the two mutations are on the same chromosome, the progeny genotypes would be 1532^I/T^1534^F/F^ and 1532^ T/T^1534^F/S^ (Fig. 3I). The F1 genotypes were 1532^T/T^1534^F/F^ and 1532^I/T^1534^F/S^, indicating that the two mutations of the parents, 1532^I/T^1534^F/S^, originated from complementary homologous chromosomes (Fig. 3I). Since no genotype of 1532^I/T^1534^F/F^ has yet been found in Lab-R, it is proposed that I1532T arose independently of F1534S during the evolution of resistance to deltamethrin, resulting in the I1532T mutation being accompanied always by F1534S in the heterozygous genotype 1532^I/T^1534^F/S^.

### Metabolic resistance with minimal fitness cost plays an important role in the later stage of resistance evolution

We observed that R30-M (RR_50_ = 34.0, adult mortality = 38.6%) with metabolic resistance showed a higher level of resistance to deltamethrin than R30-I1532T (RR_50_ = 17.5, adult mortality = 40.0%) (Fig. 1C), and its fitness cost was the lowest among these resistant strains (Fig. 2K), especially with respect to the longer life span of adults (Fig. 2E, F). Because of the rapid growth and development trend of R30-M (Fig. 2A-C), male adults with 1532^I/I^1534^F/F^ genotypes emerged first in the resistant population (Lab-R30) and the proportion was higher than that of the 1532^T/T^1534^F/F^ genotype, conferring a mating advantage (Fig. 3H). At the same time, adult females with 1532^I/I^1534^F/F^ genotypes also emerged earlier than other genotypes (Fig. 3H), giving rise to the dominant haplotype I1532/F1534 with metabolic resistance in the resistant population.

### Impact of target-site and metabolic resistance on DENV-2 susceptibility

**Fig. 4:**
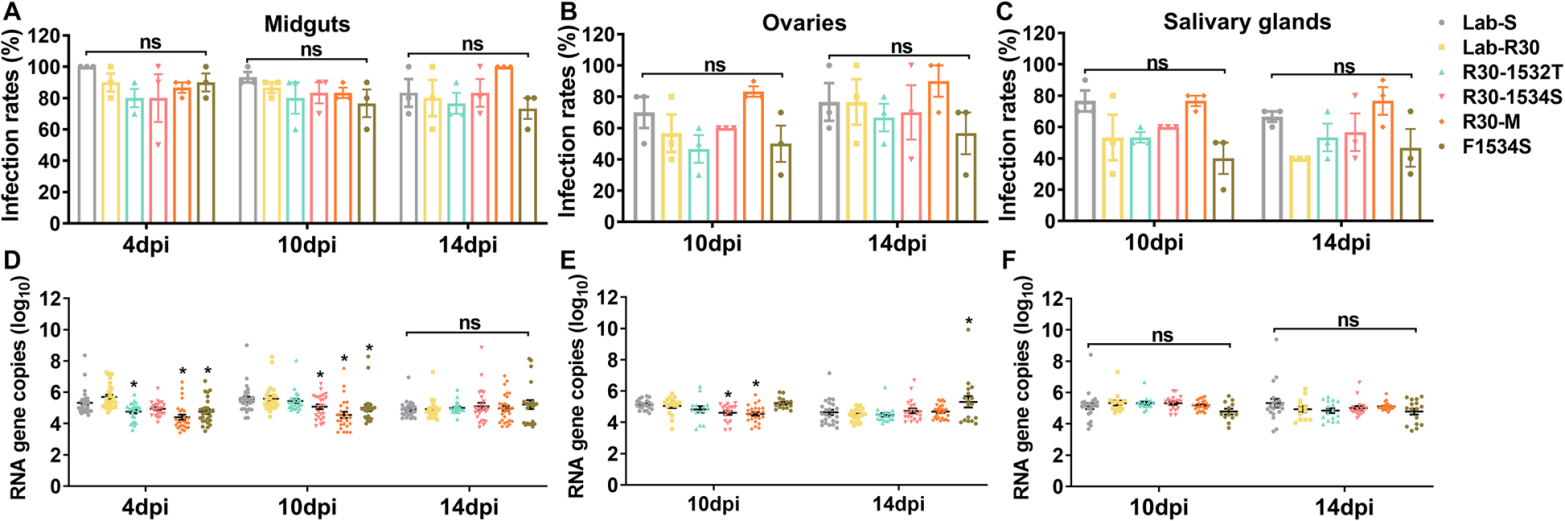
Susceptibility of DENV-2 in susceptible and resistant strains of *Aedes albopictus*. Infection rate of **A** midguts, **B** ovaries and **C** salivary glands. DENV-2 RNA copies (log_10_) in **D** infected midguts, **E** ovaries and **F** salivary glands. The midguts, ovaries and salivary glands from the six strains were dissected at 4, 10 and 14 days post-infection (dpi), and DENV-2 virus was detected by RT-qPCR. *n* = 30, 10 females at each time point with three replicates. The results are represented as the mean ± SEM. ns: not significant. *P < 0.05

To evaluate the susceptibility to DENV-2 during deltamethrin resistance evolution, 30 females of each strain were used to detect DENV-2 infection by RT-qPCR. A sample of midguts was dissected at 4 days post infection (dpi) to verify the effectiveness of oral infection. After the dengue virus extrinsic incubation period (EIP) of 8–12 days [33–36], the virus penetrates the midgut barrier and spreads to the ovaries and salivary glands, therefore, the midguts, salivary glands, and ovaries were dissected to detect DENV-2 at 4, 10 and 14 dpi. The infection rates of midgut at 4, 10 and 14 dpi were higher than 70% with no significant differences, indicating that oral infection with DENV-2 was effective (Fig. 4A; Additional File 1: Table S4). At 10 and 14 dpi, there was no significant difference in the infection rate of tissues of different strains (Fig. 4B, C). Interestingly, the infection rate of ovaries and salivary glands of Lab-R30, R30-1532T, R30-1534S and F1534S were slightly lower than that of Lab-S (Fig. 4B, C), while the infection rate of ovaries and salivary glands of R30-M were similar to Lab-S (Fig. 4C), and even slightly higher than Lab-S (Fig. 4B). The DENV-2 RNA titers in each tissue of the tested strains were 4–6 copies (log_10_)/μL at each infection time point (Fig. 4D-F). The DENV-2 virus load in midguts (R30-1532T, R30-M and F1534S at 4 dpi; R30-1534S, R30-M and F1534S at 10 dpi) and ovaries (R30-1534S and R30-M at 10 dpi) of some resistant strains were significantly lower than those of the susceptible Lab-S strain (Fig. 4D, E), and the load in F1534S ovaries was higher than that of Lab-S at 14 dpi. There was no significant difference in loads in salivary glands of tested strains at 10 and 14 dpi (Fig. 4F). Based on the positive tissue rate and virus titers at most detection time points, resistant strains may be slightly less susceptible to DENV-2 than Lab-S.

### Impacts of resistance evolution in *Aedes albopictus* on vectorial capacity

To evaluate the threat of resistance evolution in *Ae. albopictus* on dengue virus transmission, we refined the parameter *m* in Equation (2) to Equation (3), as follows: fecundity of females (*f*); development rate (*DR*); survival rate after insecticide exposure (1-*M*); and development time (*d*). The values of the parameters used are derived from the experimental data and can be used to quantitatively evaluate the VC of *Ae. albopictus* to DENV-2.

3 $${m=\frac{fDR(1-M)}{d}}$$

**Fig. 5:**
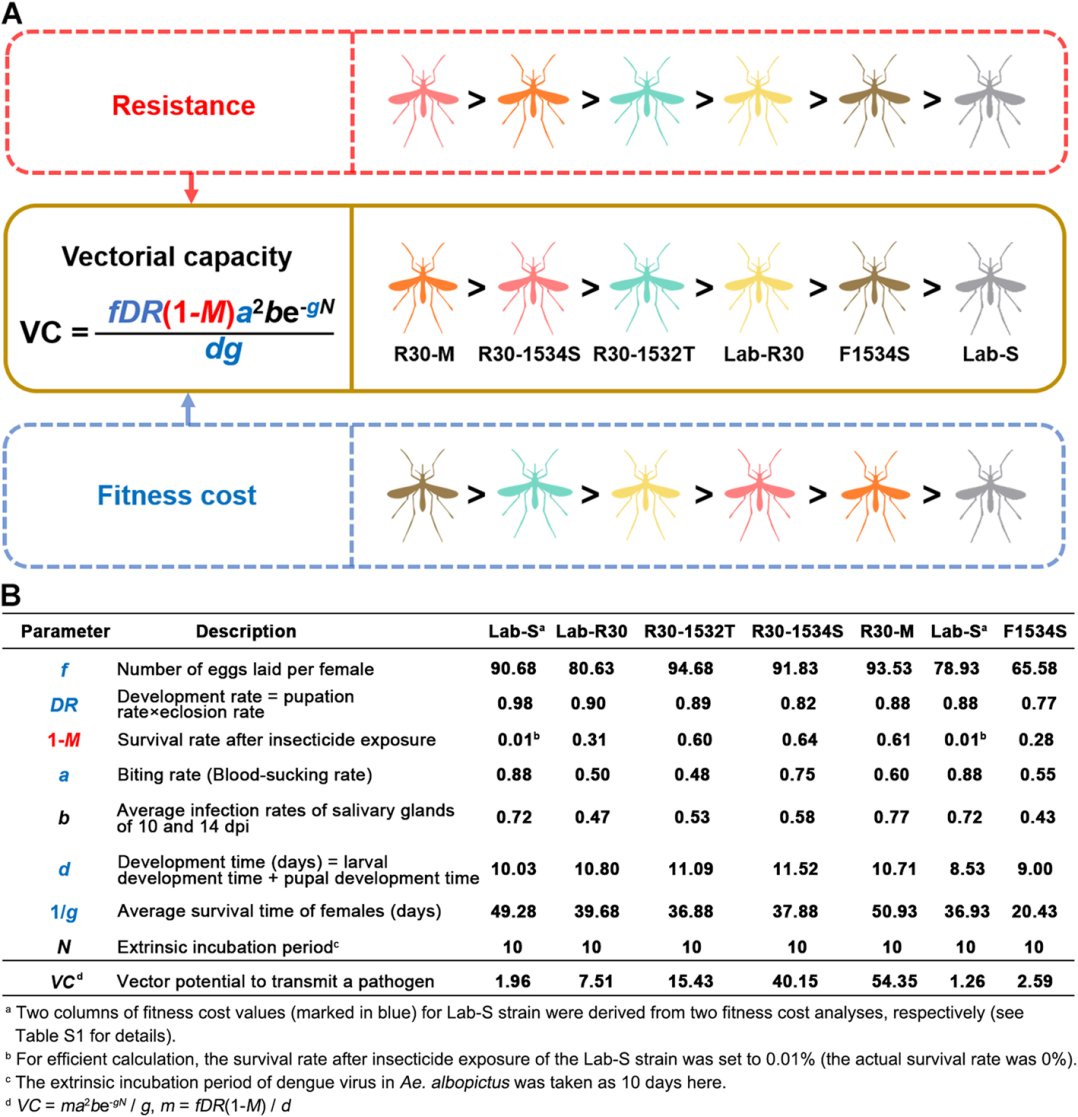
Vectorial capacity in different strains of *Ae. albopictus* during the evolution of deltamethrin resistance. **A** The impact of resistance (marked in red) and fitness cost (marked in blue) of *Ae. albopictus* were compared quantitatively by using VC equation (see Fig. 5B for details). Ranking of vectorial capacity: R30-M > R30-1534S > R30-1532T > Lab-R30 > F1534S > Lab-S. Ranking of resistance levels in VC equation: R30-1534S > R30-M > R30-1532T > Lab-R30 > F1534S > Lab-S. Ranking of fitness cost in VC equation: F1534S > R30-1532T > Lab-R30 > R30-1534S > R30-M > Lab-S. **B** Vectorial capacity of susceptible strain and resistant strains to DENV-2

We use Equation (2) and Equation (3) to calculate and compare the VC of each strain for DENV-2 with the experimentally-derived parameters (Fig. 5B) and the mortality rate of Lab-S adults at the actual 100% to 99.9% for comparison. The EIP of dengue virus in *Aedes* mosquitoes is 8–12 days [33–36], averaging 10 days at 23 ℃ and 28 ℃ [27, 37]. The results show that the VC of resistant strains was higher than that of Lab-S (Fig. 5A). Compared with Lab-S, except for R30-M with lowest fitness cost and high vector competence, other resistant strains that were inferior in the parameters related to fitness cost, including fecundity (*f*), development rate (*DR*), behavioral cost (*a*), survival (1/*g*), development time (*d*) and vector competence (*b*) could still achieve higher VC (Fig. 5A). This supports the conclusion that the survival after exposure to insecticides (1-*M*) afforded by resistance in the *m* parameter contributes greatly to the high VC of resistant strains, which is enough to overcome the disadvantage of fitness cost (Fig. 5A). With the evolution of resistance to deltamethrin in *Ae. albopictus*, the VC value of resistant populations for DENV-2 increased (Fig. 5), and R30-M dominated by metabolic resistance had the highest VC value due mainly to vector competence and minimal fitness cost.

## Discussion

The evolution of resistance alleles in pyrethroid-resistant wild *Aedes* populations is complex because of the involvement of multiple mechanisms, especially multiple *kdr* mutations and genotype combinations [12, 38–40]. In previous work [14], we constructed laboratory-derived resistant strains and identified two mechanisms involved in the evolution of deltamethrin resistance (Fig. 1A). In this study, we further constructed laboratory strains representing different resistance mechanisms (Fig. 1B). The comparisons of quantified fitness costs in these strains provide evidence for the conclusion that the heterozygous F1534S mutation (1534^F/S^), first observed in the deltamethrin-resistant population, not only confers high-level resistance, but also produces no significant fitness costs, leading to the rapid spread of resistance in the population. This is followed by the increase in frequency of homozygous F1534S (1534^S/S^) mosquitoes that have significant fitness disadvantages, prompting the emergence of an unlinked I1532T mutation with fewer side effects and a mating advantage better adapted to the selection and reproductive pressures imposed in the experiments. This is consistent with the observed frequency dynamics that the frequency of I1532T mutation began to rise after it appeared in the resistant population, while the frequency of F1534S mutation began to decline (Fig. 2C in Guo et al. [14]).

Studies have proposed that the alleles with high fitness cost are gradually replaced by the alleles with low fitness cost during the evolution of resistance [41, 42]. For example, in the process of resistance screening of *Culex pipiens pallens* collected in the field, it was observed that the frequency of L1014F mutation increased gradually, while the frequency of L1014 and L1014S mutation decreased [43]. Both I1532T and F1534S mutations have been found in deltamethrin-resistant *Aedes albopictus* population in the field [10, 44]. The impact of I1532T mutations on the phenotypes of most *Aedes albopictus* populations remains unclear, although the mutation occurs at a fairly high frequency in field populations [10]. R30-M, dominated by metabolic resistance, has almost no fitness cost, indicating that metabolic resistance may gradually become more prevalent than target-site resistance in the evolution of resistance. This is consistent with field data in regions were *Anopheles gambiae* [45] and *Culex pipiens pallens* [43] have been selected for long times by pyrethroids and support the generality of the results here.

However, it should be emphasized that other dynamics of resistance evolution may potentially occur in mosquitoes. Studies have shown that duplication of the *vgsc* gene detected in *Culex quinquefasciatus* [46] and *Aedes aegypti* [47] is thought to partially counteract the fitness cost of target mutations involved in the evolution of mosquito resistance.

The reductions in male mating success have been observed in resistant mosquitoes with target-site mutations, for example, *Ae. aegypti* with V1016G/S989P [18], *Anopheles gambiae* with L1014F [19] and *RDL*^*R*^ (dieldrin-resistant alleles) [48], and *Culex pipiens* with the *Ace. 1*^*R*^ (acetylcholinesterase-resistance alleles) [49]. Our study found that the mating competition cost of F1534S was most significant, while the resistant males with the I1532T *kdr* genotype are more successful. This is expected to directly affect the distribution of *kdr* genotypes in resistant populations, thus directing the trend of resistance evolution. This phenomenon of mating is suspected to have a greater impact on the vector population than the fecundity of females [48].

The behavioral correlation between *kdr* mutations and host-seeking ability in mosquitoes is poorly documented, except that *kdr* mutations have no significant effect on female avidity or host location in *Ae. aegypti* [18]. In addition, *An. gambiae kdr* heterozygotes (heterozygous advantage) were observed to be more active and thus quicker to locate hosts, but *kdr* homozygotes behavior was not changed significantly [20, 50]. We show here that the host-seeking ability of deltamethrin-resistant *Ae. albopictus* with *kdr* mutations is slightly lower than the susceptible strain, especially for F1534S homozygotes (F1534S), which was reduced significantly. Interestingly, both R30-1534S and F1534S are homozygous for the F1534S mutation, but R30-1534S with metabolic resistance had a stronger host-seeking ability, and its cost of growth and development also was smaller than F1534S. It is possible that the participation of metabolic enzymes can be compensatory and gradually lead to the replacement of the *kdr*-bearing mosquitoes with the greater negative pleiotropic effects resulting from the resistance mechanism. However, it should be emphasized that the gene-edited homozygous (F1534S) is far more inbred than the other selected lines, which may result in behavioral costs.

For the succession in the evolution of resistance of the two *kdr* mutations observed in previous work [14], we experimentally verified that emergence of the F1534S mutation preceded that of I1532T and that these were likely independent events as they occurred on different chromosomes of the homologous pair. Field studies also have provided evidence that pyrethroid resistance requires the sequential evolution of *kdr* mutations in *Ae. aegypti*, with multiple F1534C mutations [38, 51], and the co-existence of I1532T and F1534S mutations has been reported in wild populations of *Ae. albopictus* in China [44].

Using the vectorial capacity concept can help further evaluate the comprehensive effects of vector density control failures, the fitness costs [52, 53] and reduced vector competence [21, 22] on public health. VC is usually used in field research of vector surveillance to deduce the transmission models of vector-borne infectious diseases [23, 27, 28]. Although the life-history characteristics of vectors can be obtained in field studies, it is difficult to assess the vector competence (*b*) for arboviruses and this can be done easily in the laboratory. Previous laboratory studies have assessed the impact of factors, such as different mosquito species [29], larval competition [30] and *Beauveria bassiana* infection [31], on VC. Although, the different vector competence (*b*) caused by these factors were assessed directly, other parameters were derived from other field studies and, the comprehensive impact of these factors on VC cannot be determined.

We further refined here the parameters of the VC equation and using experimental data, found that the evolution of resistance promotes VC. This is consistent with some of the studies reviewed by Juache-Villagran et al*.* [24]. For vector competence (*b*), resistant strains with significant fitness cost were observed with low infection rates, which is consistent with our previous laboratory study [21], and Stephenson et al. [54] also found that field populations with the highest frequency of *kdr* mutations (V1016L/F1534C) had lower vector competence of DENVs. It is worth mentioning that only a few investigations describe the correspondence between field and laboratory studies [24]. In addition, R30-M with the lowest fitness cost exhibited a high susceptibility to DENV-2, further confirming our previous inference that the lower vector competence of the resistant population was due to the lack of available resources for the vector to fight against viral infection caused by the fitness cost [21]. Although the fitness cost of resistant strains reduced their VC value, the high vector density after insecticide exposure mitigates the situation, supporting the conclusion that the control of vector density should still be the primary goal of vector-borne disease control programs.

## Conclusions

Based on deltamethrin screening in the laboratory, this study revealed a possible mechanism for the evolution of deltamethrin resistance in *Aedes albopictus*. We conclude that the early stages of deltamethrin insecticide resistance evolution in *Ae. albopictus* are characterized by the emergence of target-site mutations accompanied by significant genetic loads impacting fitness in mutant homozygotes. These loads are tolerated because of the survival of the mutant mosquitoes compared to susceptible wild-types under insecticide selection. Concomitantly, the vector competence for Dengue 2 virus is decreased, but the rapid spread of resistant mosquitoes that maintain high vector densities offsets any potential beneficial effects of reduced VC. Furthermore, the progressive evolution of resistance results in mosquitoes with both target-site and metabolic resistance mutations with lower fitness costs, and this then results in mosquito populations with both high resistance levels and vectorial capacity. Thus, resistance evolution will gradually increase the threat to public health. The discovery of this evolutionary mechanism cautions us that vectors will be selected to reduce fitness costs to adapt to the insecticide pressure. Public health officials rely on a certain type of pesticides for long periods of time; therefore, the rotation of insecticides and the development of new insecticides are critical to continued control.

## Methods

### Mosquito strains

The laboratory susceptible strain (Lab-S), provided by the Shanghai Center for Disease Control and Prevention, was used as a reference strain for the resistance bioassays. The laboratory-derived resistance strain (Lab-R) was selected with deltamethrin (94.6%, Chinese Centers for Disease Control and Prevention) for 30 generations (Lab-R30) from the Lab-S strain in the previous study [14]. The F1534S mutant homozygous (F1534S, RR_50_ = 17.0 and adult mortality = 71.9%) and heterozygous (F1534S’, RR_50_ = 16.0 and adult mortality = 81.3%) strains with high deltamethrin resistance, as well as the mutant-restored strain (S1534, RR_50_ = 1.5 and adult mortality = 100.0%) with deltamethrin susceptibility, were constructed in a previous studies (Additional File 1: Fig. S2A) [14]. All three strains were derived from the Lab-S strain. All mosquito strains were reared in the standard insectary conditions (26 ± 2˚C, 14 h:10 h light/dark period, 70 ± 10% relative humidity) without insecticide exposure [21]. Non-blood-fed female adults aged 3–5 days post-eclosion (PE) and 3–4-instar larvae were used for resistance tests.

### Bioassays and synergists

The larval resistance bioassays were conducted using industrial-grade deltamethrin (94.6%, Chinese Centers for Disease Control and Prevention) following WHO guidelines [55], and the adult resistance bioassays were performed using 0.05% deltamethrin insecticide-impregnated papers (Universiti Sains Malaysia, Penang, Malaysia) following the standard WHO tube test protocol [32, 56]. The CDC (Centers for Disease Control and Prevention) bottle bioassay was used to determine the involvement of metabolic enzymes in resistance [57]. The effects of the cytochrome P450 monooxygenases (CYPs), choline/carboxylesterases (CCEs) and the glutathione-S-transferase (GSTs) on the resistance of laboratory strains to deltamethrin was tested using the synergistic agent piperonyl butoxide (PBO) (400μg per bottle), S.S.S-tributlyphosphorotrithioate (DEF) (125μg per bottle) and diethyl maleate (DM) (80μg per bottle), using the protocol described by CDC [57]. The significantly increased mortality of adult mosquitoes after the use of synergists likely results from the synergists mitigating the apparent resistance observed in the CDC bottle bioassay, and the corresponding inhibited detoxification enzymes play a role in that particular resistance mechanism. More details can be found in Guo et al. [14].

### DNA extraction and *kdr* mutation detection

Genomic DNA was extracted from the legs of individual adult females using the REDExtract-N-Amp™ Tissue PCR Kit (Sigma Aldrich). Diagnostic nucleotide sequences in domains II (480 bp), III (346 bp) and IV (280 bp), encompassing the amino acids 989, 1011, 1014, 1016, 1534 and 1763 of the *vgsc* gene (the numbering is based on the *Musca domestica vgsc* gene and is consistent with previously-established *knockdown resistance* (*kdr)* codon nomenclature), were amplified by PCR [58]. Amplicons were purified with the MiniBEST DNA Fragment Purification Kit (Takara) and sequenced directly. Sequence analysis and alignments were performed with Chromas software and MEGA7 (version 7.1.0, http://www.megasoftware.net/).

### Isolation of sub-strains from the Lab-R30 strain

The genotypes of unmated adult male and female mosquitoes of the Lab-R30 strain were identified by extracting genomic DNA from mosquito legs as described earlier. The unmated individuals with the same genotypes were mated to obtain the R30-1532T (1532^T/T^1534^F/F^), R30-1534S (1532^I/I^1534^S/S^) and R30-M (1532^I/I^1534^F/F^) strains with metabolic resistance (Fig. 1B and Fig. S1).

### Fitness cost analysis

All experiments of fitness cost were performed in the standard insectary conditions (26 ± 2˚C, 14 h:10 h light/dark period, 70 ± 10% relative humidity).

#### Egg hatchability

100 eggs were collected from wet filter paper 5 days after the females laid eggs, and placed in water for incubation. The number of larvae hatched was recorded after one week, with four replicates per strain.

#### Life table at larvae stage

The eggs of different strains were incubated in water for 24h at the same time to hatch for 24 h. Three replicates per strain of 50 newly hatched larvae were then transferred to plastic bowl with 300mL dechlorinated water and ground turtle food. The number of pupae and eclosed males and females were recorded daily.

#### Adult mosquito survival

Newly emerged (24h PE) males and females were separated and transferred to paper bowls covered with gauze (a bowl of 40 mosquitoes per strain). A 10% glucose solution was supplied and dead adults were recorded daily until all died.

#### Female mosquito fecundity

Newly-emerged (24h PE) adults were mated in cages for 2–3 days, and the females (3-4d PE) were provided defibrinated sheep blood after starvation for 24 h. After cold-anesthetization, 40 fully engorged females from each strain were placed individually into 250mL paper cups with filter paper. Water was added three days later to the paper cup to moisten the filter paper and induce females to lay eggs. The number of eggs/female were counted after five days.

#### Measurements of adult mosquito size

The sizes of adult mosquitoes were recorded by wing length and body weight. Control and experimental samples of adult mosquitoes were raised from eggs at the same time and under the same conditions. Newly-emerged (24h PE) adults were mated in cages for 2–3 days, and then the wings of adult mosquitoes (3-4d PE) were removed and viewed with a microscope connected to a computer and camera for photography. Image-pro Plus software was used to measure the wing length defined as the distance from the axillary incision to the apical margin (excluding fringes). The wing length of an adult is represented by the average of both its wings. Each strain has 30 samples of wing length for both females and males. Adult mosquitoes were dried in an oven at 60 ℃ for four hours and their weight measured by an electronic analytical balance in groups of eight (four groups per strain).

### Molecular modeling of wild type and mutated protein VGSC

The wild-type (VGSC_WT) models of VGSC proteins were computed by the SWISS-MODEL [59] server homology modelling pipeline in a previous study [14]. The F1534S and I1532T mutations were performed in MOE v2018.0101. The structures of the proteins (WT, F1534S and I1532T) were optimized by MD simulation using AMBER16 [60]. MOE Dock was used for molecular docking of proteins with deltamethrin, as detailed in the previous study [14].

### Male mating success

Thirty unmated (24h PE) males and females (1:1 ratio) each from R30-1532T and R30-1534S were transferred to the same cage. After mating for 2–3 days, females were allowed to lay eggs in separate cups after a blood meal, and the offspring of a single female were hatched in water. Genotypes of females and their offspring were detected to determine the genotype of males that successfully mated with them. Three replicates were performed.

### Host-seeking behavior

Mosquitoes 3–4 days post-eclosion (PE) were starved for 24 h and pools of 10 females were transferred into a rearing cage, allowed to rest for 10 min post-transfer, and then offered a blood meal from the forearm (exposed area 4.5 cm × 16.0 cm) of a human adult volunteer for five min between 1700 and 1730 h [18]. The host-locating time (time from exposure to arm to initiation of probing) and number of engorged females were recorded. Four replicates of 10 mosquitoes from each strain were used for the experiments and offered blood from the arm of the same human volunteer.

### Vector competence

#### Cell line and virus

C6/36 cells were cultured in RPMI-1640 medium supplemented with 10% heat inactivated fetal bovine serum (FBS) and maintained at 28 ℃. Dengue virus 2 (DENV-2, New Guinea C, GenBank: AF038403.1) was provided by the Key Laboratory of Tropical Disease Control of Sun Yat-sen University (Guangzhou, China). The supernatant of DENV-2 was harvested after enrichment in C6/36 cells at 37 ℃ for 36–48 h until obvious cytopathic effects were observed and then stored at –80 ℃.

#### Oral infections of mosquitoes

DENV-2 supernatant (10^4^ pfu/mL) was mixed with defibrinated sheep blood at a ratio of 2:1, maintained at 37˚C for 30 min and then transferred to a Hemotek blood reservoir unit (Discovery Workshops, L0061ncashire, United Kingdom). Females aged 3–5 days PE were starved for 24 h and allowed to feed on the infectious blood meal for 30 min. After cold-anesthetization, fully engorged mosquitoes were selected and placed into 250-mL paper cups covered with gauze (10 mosquitoes/cup). All treatments were maintained at 28 ℃, 80% relative humidity and a light: dark cycle of 16 h:8 h and adults fed on 10% glucose water. Midguts were dissected at 4, 10 and 14 days post infection (dpi), and ovaries and salivary glands were dissected to detect DENV-2 at 10 and 14 dpi (10 females at each time point with three replicates). Mosquito infections were conducted in a Biological Safety Level 2 lab.

#### DENV-2 detection and quantification

Total RNA of collected tissues were extracted according to TRIzol manufacturer’s protocol (Ambion, Life Technologies, Carlsbad, CA, United States) and dissolved in 20 µl of RNase-free water. cDNA synthesis was performed by using the GoScript Reverse Transcription System (Promega, Madison, WI, USA) with random primers. Absolute quantitative real-time PCR (RT-qPCR) was used to quantify the DENV-2 RNA copies of samples following previous protocol [21]. A standard curve for DENV-2 detection was established by tenfold dilutions of the plasmid standard (1.82 × 10^3^–1.82 × 10^8^copies/μL) constructed as previously described [37]. Each sample was conducted in three replicates, and the results were determined by the melting curve and cycle threshold values. The vector competence of the *Ae. albopictus* mosquitoes was evaluated by calculating the infection rate of tissues (no. infected tissues/no. tested mosquitoes).

### Statistical analysis

The extent of resistance in larval bioassays was measured by the resistance ratio (RR_50_), which is calculated as the ratio of LC_50_ for test strains to the LC_50_ of the Lab-S strain. LC_50_ were estimated using the log-probit models. Larval resistance status was defined as susceptible if RR_50_ < 5, moderately resistant if 5 < RR_50_ < 10, and highly resistant if RR_50_ > 10 [32]. For adult bioassays, resistant status definition follows the WHO classification criteria: resistant if mortality < 90%, probably resistant if mortality was between 90 and 98%, and susceptible if mortality > 98% [32, 56]. For CDC bottle bioassays, 98% – 100% mortality is classified as susceptible, 80% – 97% mortality as possible resistant, < 80% mortality as resistant [57]. Chi-square test was used to examine differences in adult mortality rates between synergist-control groups and synergist-exposed groups.

The differences between the strains in average development time, female fecundity, wing length, body weight, host-locating time and DENV-2 RNA copies (log_10_) were evaluated by One-Way ANOVA, followed by Student–Newman–Keuls (SNK) test. The difference in the survival time of adult mosquitoes was determined by Kaplan–Meier survival analysis and log-rank test (GraphPad Prism 7). Chi-square test was used to compare developmental rate, hatching rate, mating rate, blood-sucking rate and DENV-2 infection rate of difference strains. All statistical analyses were performed with SPSS 20.0 (IBM) and *P* < 0.05 was considered statistically significant.

### Supplementary Information

**Additional file 1:** **Figure S1.** Metabolic resistances to deltamethrin in different* Ae. albopictus* strains with varied genotypes at 1532 and 1534 sites. (A) Metabolic resistance in different strains. The synergistic agents, piperonyl butoxide (PBO), S.S.S-tributlyphosphorotrithioate (DEF) and diethyl maleate (DM), are respective inhibitors of cytochrome P450 monooxygenases (P450s), choline/carboxylesterases (CCEs) and glutathione-S-transferase (GSTs). Adults treated with deltamethrin were used as control. The error bars represent the means ± SEM of five replicates. Different lowercase letters (a and b) indicate significant difference (*P* < 0.001) based on Chi-square test. (B) Genotypes of 1532 and 1534 sites of *vgsc* gene in different strains. Representative chromatograms of direct sequencing of the PCR products for genotyping 1532 and 1534 sites. Strains used to represent different resistance mechanisms: Lab-S (susceptible reference strain), F1534S’ (1532^I/I^1534^F/S^), F1534S (1532^I/I^1534^S/S^), Lab-R30 (I1532T/F1534Sand metabolic resistance), R30-1534S (1532^I/I^1534^S/S^ and metabolic resistance), R30-1532T (1532^T/T^1534^F/F^ and metabolic resistance) and R30-M (metabolic resistance). **Figure S2. **Fitness cost of deltamethrin resistance in *Aedes albopictus* larvae and adults caused by F1534S mutation. (A) An F1534S strain with the F1534S homozygous mutation was constructed from a susceptible Lab-S strain by CRISPR/Cas9, and a “restored” susceptible S1534F strain was constructed by backcrossing of the F1534S with Lab-S. The mutant heterozygote, F1534S’, was obtained by crossing Lab-S with the F1534S strain [14]. Temporal trend of pupation rate (B) and eclosion rate (C). (D) Larval and pupal developmental time. (E) Pupation rate of larvae and eclosion rate of pupae. B-E*n *= 3, 50 newly hatched larvae per strain in each experiment. (F) Survival curves of female adults (*n *= 40). Comparison of survival curves between different strains and Lab-S strain base on Log-rank test: F1534S vs Lab-S (*χ*^2^=71.12, *df*=1, *P *< 0.0001), F1534S’ vs Lab-S (*χ*^2^=1.146, *df*=1,*P *= 0.2844), S1534F vs Lab-S (*χ*^2^=0.4271, *df*=1, *P *= 0.5314). (G) Survival curves of male adults (*n *= 40). Comparison of survival curves between different strains and Lab-S strain base on Log-rank test: F1534S vs Lab-S (*χ*^2^=48.65, *df*=1, *P *< 0.0001), F1534S’ vs Lab-S (*χ*^2^=1.476, *df*=1,*P *= 0.2243), S1534F vs Lab-S (*χ*^2^=5.079, *df*=1, *P *= 0.0242). (H) Weight of adults (*n *= 4, 8 pooled adults each experiment). (I) Wing length of adults (*n *= 30). (J) Fecundity of female adults (*n *= 40). (K) Hatching rate per 100 eggs (*n *= 4). (L) Comprehensive quantification of fitness cost. Population vigor = (number of eggs*hatching rate*pupation rate*eclosion rate*average life span of adults*average weight of adults*average wing length of adults)/(larval development time + pupal development time). Ranking of fitness cost of several strains: F1534S > S1534F > Lab-S > F1534S’. The results are represented as the mean ± SEM. ns: not significant. Different lowercase letters, a and b, represent significant differences (*P* < 0.05).** Table S1. **Life table values of different strains of *Aedes albopictus* under laboratory conditions. **Table S2. **Results of one-way ANOVA, log-rank test and Chi-square test on life history traits. **Table S3. **The percentage and time (seconds) of starved females from each strain successfully taking a human blood meal in 5 minutes. **Table S4. **DENV-2 infection rates of tissues in susceptible and resistant strains of *Aedes albopictus*.

**Additional file 2:** **Data S1.** Excel spreadsheet containing, in separate sheets, the numerical values that were used to generate graphs, histograms etc. for Figure panels 1C, 2A-K, 3G, 3H, 3J, 3K, 4A-F, 5B, S1A and S2B-L.

**Additional file 3:** **Data S2.** The structures of the mutant proteins I1532T performed in this study were optimized by MD simulation using AMBER16.

## Data Availability

All data generated or analyzed during this study are included in this published article and its supplementary information files. Original data of life table values, host seeking behavior and DENV-2 infection rates of different *Ae. albopictus* strains can be found in Additional file 1. Original data of deltamethrin resistances, fitness, mating success, host seeking behavior, susceptibility and vectorial capacity to DENV-2 in different *Ae. albopictus* strains are provided in Additional file 2. The structures of the mutant proteins I1532T optimized by molecular dynamics simulation can be found in Additional file 3.

## Abbreviations

*Ae albopictus*: *Aedes albopictus*
*Ae. aegypti*: *Aedes aegypti*
*An. gambiae*: *Anopheles gambiae*
*kdr*: Knockdown resistance
*vgsc gene*: *Voltage gated sodium channel gene*
VGSC: Voltage gated sodium channel
F1534S: Phenylalanine-to-serine substitution at position 1534 in VGSC
I1532T: Isoleucine-to-threonine substitution at position 1532 in VGSC
DENV: Dengue virus
DENV-2: Dengue virus type-2
Lab-R: An *Ae. albopictus* laboratory deltamethrin-resistant strain
Lab-S: An *Ae. albopictus* laboratory susceptible strain
P450s: Cytochrome P450 monooxygenase
CCEs: Choline/carboxylesterases
GSTs: Glutathione-S-transferases
VC: Vectorial capacity
EIP: Extrinsic incubation period
RR_50_: Resistance ratio
RMSD: Root-means-square deviation
dpi: Days post infection
L1014F: Leucine-to-phenylalanine substitution at position 1014 in VGSC
L1014S: Leucine-to-serine substitution at position 1014 in VGSC
V1016G: Valine-to-glycine substitution at position 1016 in VGSC
S989P: Serine-to-proline substitution at position 989 in VGSC
*RDL*^*R*^: Dieldrin-resistant alleles
*Ace. 1*^*R*^: Acetylcholinesterase-resistance alleles
F1534C: Phenylalanine-to-cysteine substitution at position 1534 in VGSC
V1016L: Valine-to-Leucine substitution at position 1016 in VGSC
PE: Post-eclosion
WHO: World Health Organization
CDC: Centers for Disease Control and Prevention
CYPs: Cytochrome P450 monooxygenases
PBO: Piperonyl butoxide
DEF: S.S.S-tributlyphosphorotrithioate
DM: Diethyl maleate
FBS: Fetal bovine serum
SNK: Student-Newman-Keuls
