# Supplementary material for: The dynamics of deltamethrin resistance evolution in Aedes albopictus has an impact on fitness and dengue virus type-2 vectorial capacity
Source: BMC Biol. 2023 Sep 13;21:194. doi: 10.1186/s12915-023-01693-0 (PMC10500878; doi:10.1186/s12915-023-01693-0)
Supplement: Supplementary file 1 — Additional file 1: Figure S1. Metabolic resistances to deltamethrin in different Ae. albopictus strains with varied genotypes at 1532 and 1534 sites. (A) Metabolic resistance in different strains. The synergistic agents, piperonyl butoxide (PBO), S.S.S-tributlyphosphorotrithioate (DEF) and diethyl maleate (DM), are respective inhibitors of cytochrome P450 monooxygenases (P450s), choline/carboxylesterases (CCEs) and glutathione-S-transferase (GSTs). Adults treated with deltamethrin were used as control. The error bars represent the means ± SEM of five replicates. Different lowercase letters (a and b) indicate significant difference (P < 0.001) based on Chi-square test. (B) Genotypes of 1532 and 1534 sites of vgsc gene in different strains. Representative chromatograms of direct sequencing of the PCR products for genotyping 1532 and 1534 sites. Strains used to represent different resistance mechanisms: Lab-S (susceptible reference strain), F1534S’ (1532I/I1534F/S), F1534S (1532I/I1534S/S), Lab-R30 (I1532T/F1534Sand metabolic resistance), R30-1534S (1532I/I1534S/S and metabolic resistance), R30-1532T (1532T/T1534F/F and metabolic resistance) and R30-M (metabolic resistance). Figure S2. Fitness cost of deltamethrin resistance in Aedes albopictus larvae and adults caused by F1534S mutation. (A) An F1534S strain with the F1534S homozygous mutation was constructed from a susceptible Lab-S strain by CRISPR/Cas9, and a “restored” susceptible S1534F strain was constructed by backcrossing of the F1534S with Lab-S. The mutant heterozygote, F1534S’, was obtained by crossing Lab-S with the F1534S strain [14]. Temporal trend of pupation rate (B) and eclosion rate (C). (D) Larval and pupal developmental time. (E) Pupation rate of larvae and eclosion rate of pupae. B-En = 3, 50 newly hatched larvae per strain in each experiment. (F) Survival curves of female adults (n = 40). Comparison of survival curves between different strains and Lab-S strain base on Log-rank test: F1534S vs Lab [file 12915_2023_1693_MOESM1_ESM.pdf]

Supporting Information for

The dynamics of deltamethrin resistance evolution in *Aedes albopictus* has an impact on fitness and dengue virus type-2 vectorial capacity

Authors: Yijia Guo[1], Ke Hu[1], Jingni Zhou[1], Zhensheng Xie[1], Yijie Zhao[1], Siyu Zhao[1], Jinbao Gu[1], Xiaohong Zhou[1], Guiyun Yan[2], Anthony A. James[3,4,*], and Xiao-Guang Chen[1,*]

Affiliations:

[1]Department of Pathogen Biology, Institute of Tropical Medicine, School of Public Health, Southern Medical University, Guangzhou, China

[2]Program in Public Health, University of California, Irvine, Irvine, CA, USA

[3]Department of Microbiology & Molecular Genetics, University of California, Irvine CA USA 92697-4025

[4]Department of Molecular Biology & Biochemistry, University of California, Irvine CA USA 92697-3900

*Corresponding author. Email: xgchen@smu.edu.cn or aajames@uci.edu.

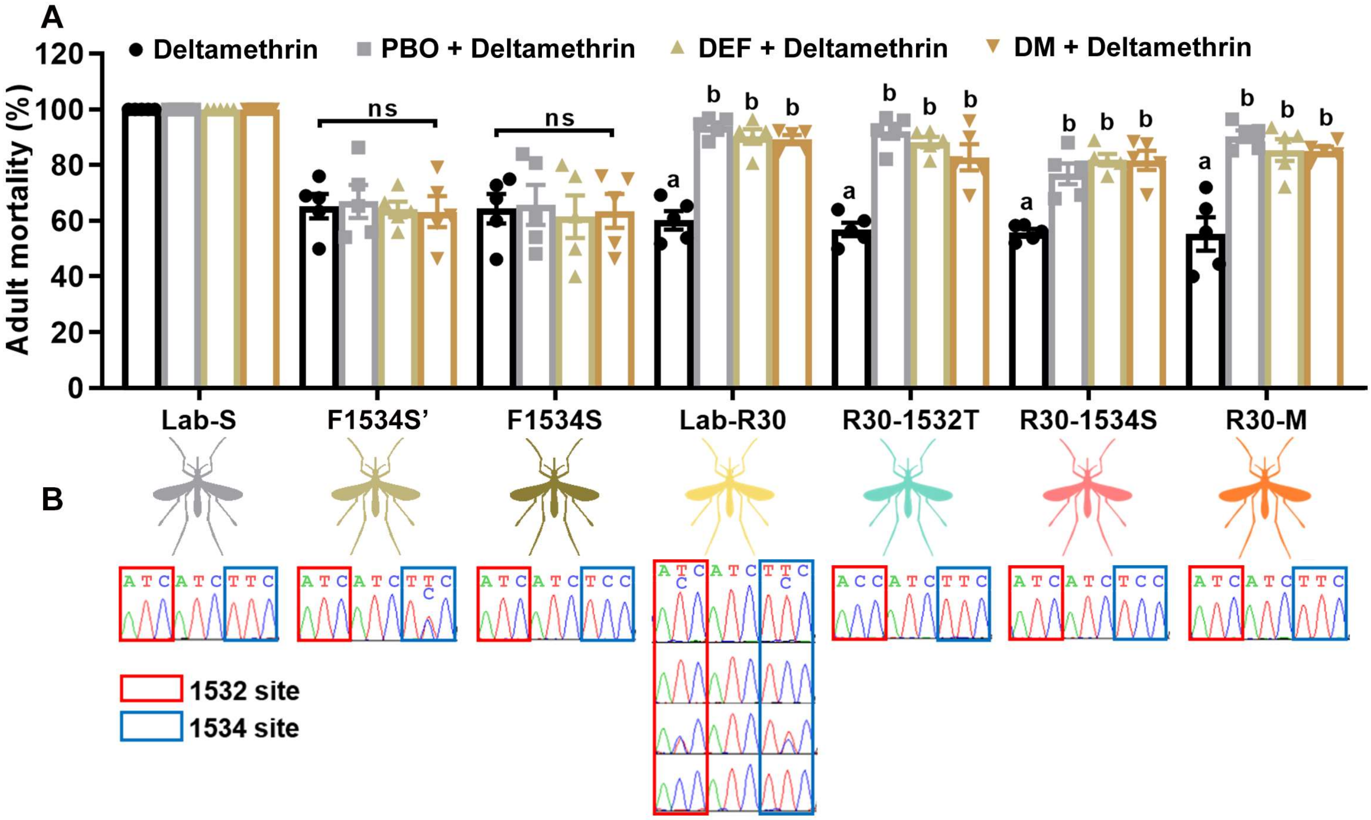

Figure S1. Metabolic resistances to deltamethrin in different *Ae. albopictus* strains with varied genotypes at 1532 and 1534 sites. (A) Metabolic resistance in different strains. The synergistic agents, piperonyl butoxide (PBO), S.S.S-tributlyphosphorotrithioate (DEF) and diethyl maleate (DM), are respective inhibitors of cytochrome P450 monooxygenases (P450s), choline/carboxylesterases (CCEs) and glutathione-S-transferase (GSTs). Adults treated with deltamethrin were used as control. The error bars represent the means ± SEM of five replicates. Different lowercase letters (a and b) indicate significant difference ($P < 0.001$) based on Chi-square test. (B) Genotypes of 1532 and 1534 sites of *vgsc* gene in different strains. Representative chromatograms of direct sequencing of the PCR products for genotyping 1532 and 1534 sites. Strains used to represent different resistance mechanisms: Lab-S (susceptible reference strain), F1534S' ($1532^{I/I}1534^{F/S}$), F1534S ($1532^{I/I}1534^{S/S}$), Lab-R30 (I1532T/F1534S and metabolic resistance), R30-1534S ($1532^{I/I}1534^{S/S}$ and metabolic resistance), R30-1532T ($1532^{T/T}1534^{F/F}$ and metabolic resistance) and R30-M (metabolic resistance).

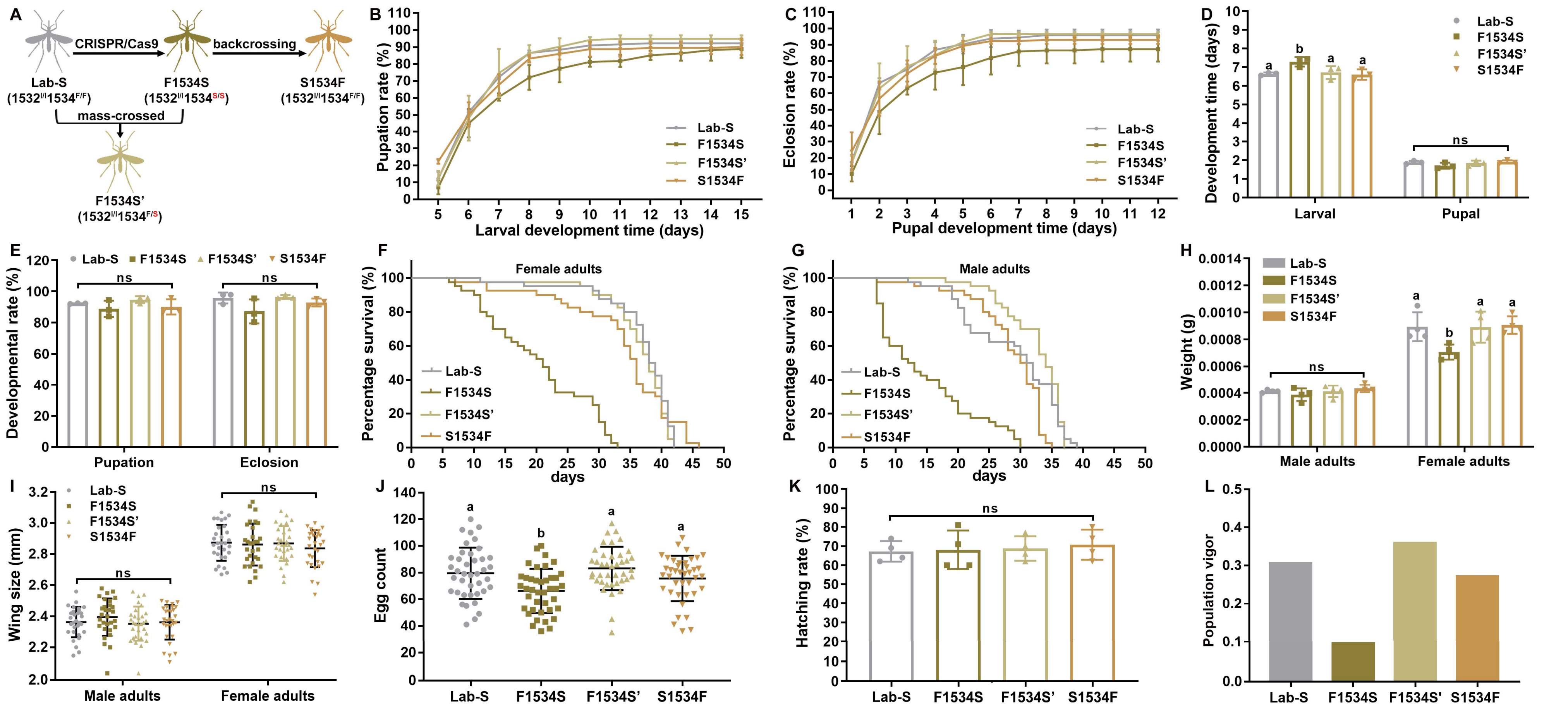

Figure S2. Fitness cost of deltamethrin resistance in *Aedes albopictus* larvae and adults caused by F1534S mutation. (A) An F1534S strain with the F1534S homozygous mutation was constructed from a susceptible Lab-S strain by CRISPR/Cas9, and a "restored" susceptible S1534F strain was constructed by backcrossing of the F1534S with Lab-S. The mutant heterozygote, F1534S', was obtained by crossing Lab-S with the F1534S strain [14]. Temporal trend of pupation rate (B) and eclosion rate (C). (D) Larval and pupal developmental time. (E) Pupation rate of larvae and eclosion rate of pupae. B-E $n = 3$, 50 newly hatched larvae per strain in each experiment. (F) Survival curves of female adults ($n = 40$). Comparison of survival curves between different strains and Lab-S strain base on Log-rank test: F1534S vs Lab-S (χ^2=71.12, *df*=1, $P < 0.0001$), F1534S' vs Lab-S (χ^2=1.146, *df*=1, $P = 0.2844$), S1534F vs Lab-S (χ^2=0.4271, *df*=1, $P = 0.5314$). (G) Survival curves of male adults ($n = 40$). Comparison of survival curves between different strains and Lab-S strain base on Log-rank test: F1534S vs Lab-S (χ^2=48.65, *df*=1, $P < 0.0001$), F1534S' vs Lab-S (χ^2=1.476, *df*=1, $P = 0.2243$), S1534F vs Lab-S (χ^2=5.079, *df*=1, $P = 0.0242$). (H) Weight of adults ($n = 4$, 8 pooled adults each experiment). (I) Wing length of adults ($n = 30$). (J) Fecundity of female adults ($n = 40$). (K) Hatching rate per 100 eggs ($n = 4$). (L) Comprehensive quantification of fitness cost. Population vigor = (number of eggs*hatching rate*pupation rate*eclosion rate*average life span of adults*average weight of adults*average wing length of adults)/(larval development time + pupal development time). Ranking of fitness cost of several strains: F1534S > S1534F > Lab-S > F1534S'. The results are represented as the mean ± SEM. ns: not significant. Different lowercase letters, a and b, represent significant differences ($P < 0.05$).

Table S1. Life table values of different strains of *Aedes albopictus* under laboratory conditions.

Strain	Larval development time (days)	Pupal development time (days)	Pupation rates (%)	Eclosion rates (%)	Average survival time (days)		Weight (g)		Wing length (mm)		Number of eggs per female	Egg hatchability (%)
					♀	♂	♀	♂	♀	♂		
Lab-S	6.65 ± 0.04	1.88 ± 0.05	92.25 ± 0.13	95.86 ± 2.01	36.93 ± 0.99	28.90 ± 1.19	0.0009	0.0004	2.87 ± 0.02	2.36 ± 0.02	78.93 ± 2.81	67.25 ± 2.69
F1534S	7.29 ± 0.15	1.71 ± 0.09	88.89 ± 3.03	87.22 ± 4.44	20.43 ± 1.31	14.80 ± 1.22	0.0007	0.0004	2.86 ± 0.02	2.39 ± 0.02	65.58 ± 2.44	67.00 ± 5.05
F1534S’	6.72 ± 0.20	1.84 ± 0.08	94.79 ± 1.25	96.55 ± 0.69	36.45 ± 0.96	32.53 ± 0.74	0.0009	0.0004	2.87 ± 0.02	2.35 ± 0.02	82.71 ± 2.36	68.75 ± 3.20
S1534F	6.61 ± 0.16	1.91 ± 0.05	90.19 ± 2.82	92.99 ± 1.46	33.85 ± 1.44	28.45 ± 0.95	0.0009	0.0004	2.86 ± 0.02	2.36 ± 0.02	74.00 ± 2.53	70.75 ± 1.74
Lab-S	7.73 ± 0.21	2.30 ± 0.04	99.29 ± 0.71	99.33 ± 0.67	49.28 ± 1.38	35.30 ± 1.08	0.0011	0.0004	2.92 ± 0.02	2.30 ± 0.03	90.68 ± 2.82	67.25 ± 2.69
Lab-R30	8.50 ± 0.06	2.30 ± 0.09	93.11 ± 2.16	96.61 ± 0.66	39.68 ± 1.51	26.85 ± 1.65	0.0011	0.0004	2.91 ± 0.02	2.28 ± 0.03	80.63 ± 4.24	68.25 ± 9.42
R30-1532T	8.61 ± 0.19	2.48 ± 0.01	89.35 ± 4.26	100 ± 0.00	36.88 ± 0.81	27.03 ± 1.80	0.0011	0.0004	2.81 ± 0.02	2.28 ± 0.03	94.68 ± 3.47	69.00 ± 1.29
R30-1534S	9.50 ± 0.29	2.02 ± 0.31	84.77 ± 2.90	96.93 ± 2.06	37.88 ± 1.08	22.18 ± 1.39	0.0009	0.0004	2.81 ± 0.03	2.27 ± 0.03	91.83 ± 2.57	71.25 ± 2.46
R30-M	8.54 ± 0.13	2.17 ± 0.04	91.80 ± 2.22	96.27 ± 2.67	50.93 ± 1.56	36.50 ± 1.68	0.0011	0.0004	2.92 ± 0.02	2.31 ± 0.02	93.53 ± 2.97	67.25 ± 4.33

Values are means ± standard error.

Table S2. Results of one-way ANOVA, log-rank test and Chi-square test on life history traits.

Strains	indicators	χ^2	F	*df*	*P*
Lab-S<br>F1534S<br>F1534S'<br>S1534F	Larval development time (days)*	-	4.586	3	0.038
	Pupal development time (days)	-	1.656	3	0.253
	Pupation rates (%)	1.253	-	3	0.740
	Eclosion rates (%)	3.819	-	3	0.282
	Life span of female*	137.8	-	3	< 0.0001
	Life span of male*	129.2	-	3	< 0.0001
	Weight of female adult*	-	4.570	3	0.023
	Weight of male adult	-	1.190	3	0.355
	Wing length of female adult	-	0.546	3	0.652
	Wing length of male adult	-	0.847	3	0.471
	Number of eggs per female*	-	7.052	3	< 0.001
	Egg hatchability (%)	0.407	-	3	0.939
Lab-S<br>Lab-R30<br>R30-1532T<br>R30-1534S<br>R30-M	Larval development time (days)*	-	10.739	4	0.001
	Pupal development time (days)	-	1.333	4	0.323
	Pupation rates (%)	8.286	-	4	0.082
	Eclosion rates (%)	7.073	-	4	0.132
	Life span of female*	104.5	-	4	< 0.0001
	Life span of male*	79.1	-	4	< 0.0001
	Weight of female adult*	-	3.567	4	0.031
	Weight of male adult	-	0.233	4	0.916
	Wing length of female adult*	-	5.653	4	< 0.001
	Wing length of male adult	-	0.439	4	0.780
	Number of eggs per female*	-	2.945	4	0.022
	Egg hatchability (%)	0.518	-	4	0.972

* There were significant differences among the several strains compared.

Table S3. The percentage and time (seconds) of starved females from each strain successfully taking a human blood meal in 5 minutes.

Strains	Lab-S	R30	R30-1532T	R30-1534S	R30-M	F1534S
Fed (%)	87.50 ± 4.79	50.50 ± 4.08	47.50 ± 7.50	75.00 ± 5.00	60.00 ± 9.13	55.00 ± 9.57
Time (s)	97.82 ± 8.56	96.43 ± 17.46	89.32 ± 5.76	67.67 ± 6.05	89.94 ± 10.74	119.39 ± 15.13

Values are means ± standard error.

Table S4. DENV-2 infection rates of tissues in susceptible and resistant strains of *Aedes albopictus*.

Tissues	Midguts*			Ovaries†		Salivary glands‡	
dpi	4dpi	10dpi	14dpi	10dpi	14dpi	10dpi	14dpi
Lab-S	100.00 ± 0.00	93.33 ± 3.33	83.33 ± 8.82	70.00 ± 10.00	76.67 ± 12.02	76.67 ± 6.67	66.67 ± 3.33
Lab-R30	90.00 ± 5.77	86.67 ± 3.33	80.00 ± 11.55	56.67 ± 12.02	76.67 ± 14.53	53.33 ± 14.53	40.00 ± 0.00
R30-1532T	80.00 ± 5.77	80.00 ± 10.00	76.67 ± 6.67	46.67 ± 8.82	66.67 ± 8.82	53.33 ± 3.33	53.33 ± 8.82
R30-1534S	80.00 ± 15.28	83.33 ± 6.67	83.33 ± 8.82	60.00 ± 0.00	70.00 ± 17.32	60.00 ± 0.00	56.67 ± 12.02
R30-M	86.67 ± 3.33	83.33 ± 3.33	100.00 ± 0.00	83.33 ± 3.33	90.00 ± 10.00	76.67 ± 3.33	76.67 ± 8.82
F1534S	90.00 ± 5.77	76.67 ± 8.82	73.33 ± 6.67	50.00 ± 11.55	56.67 ± 13.33	40.00 ± 10.00	46.67 ± 12.02

The results are represented as the mean ± standard error.

*Infection rates of midguts = no. infected midguts/no. tested midguts.

†Infection rates of ovaries = no. infected ovaries/no. tested ovaries.

‡Infection rates of salivary glands = no. infected salivary glands/no. tested salivary glands.
